# Small-fibre neuropathy in men with type 1 diabetes and erectile dysfunction: a cross-sectional study

**DOI:** 10.1007/s00125-017-4245-z

**Published:** 2017-03-29

**Authors:** Shazli Azmi, Maryam Ferdousi, Uazman Alam, Ioannis N. Petropoulos, Georgios Ponirakis, Andrew Marshall, Omar Asghar, Hassan Fadavi, Wendy Jones, Mitra Tavakoli, Andrew J. M. Boulton, Maria Jeziorska, Handrean Soran, Nathan Efron, Rayaz A. Malik

**Affiliations:** 1 Centre for Endocrinology and Diabetes, Institute of Human Development, University of Manchester and Central Manchester NHS Foundation Trust, Manchester Academic Health Science Centre, 46 Grafton Street, Core Technology Facility, Manchester, M13 9NT UK; 2 Department of Eye and Vision Science, Institute of Ageing and Chronic Disease, University of Liverpool, Liverpool, UK; 3 Department of Medicine, Weill Cornell Medicine-Qatar, Doha, Qatar; 4 Department of Clinical Neurophysiology, Central Manchester NHS Foundation Trust, Manchester, UK; 5 Department of Medicine, The University of Exeter Medical School, Exeter, UK; 6 Institute of Health and Biomedical Innovation, Queensland University of Technology, Brisbane, QLD Australia

**Keywords:** Corneal confocal microscopy, Erectile dysfunction, Neuropathy, Small-fibre neuropathy, Type 1 diabetes

## Abstract

**Aims/hypothesis:**

The aim of this study was to identify the contribution of small- and large-fibre neuropathy to erectile dysfunction in men with type 1 diabetes mellitus.

**Methods:**

A total of 70 participants (29 without and 41 with erectile dysfunction) with type 1 diabetes and 34 age-matched control participants underwent a comprehensive assessment of large- and small-fibre neuropathy.

**Results:**

The prevalence of erectile dysfunction in participants with type 1 diabetes was 58.6%. After adjusting for age, participants with type 1 diabetes and erectile dysfunction had a significantly higher score on the Neuropathy Symptom Profile (mean ± SEM 5.3 ± 0.9 vs 1.8 ± 1.2, *p* = 0.03), a higher vibration perception threshold (18.3 ± 1.9 vs 10.7 ± 2.4 V, *p* = 0.02), and a lower sural nerve amplitude (5.0 ± 1.1 vs 11.7 ± 1.5 mV, *p* = 0.002), peroneal nerve amplitude (2.1 ± 0.4 vs 4.7 ± 0.5 mV, *p* < 0.001) and peroneal nerve conduction velocity (34.8 ± 1.5 vs 41.9 ± 2.0 m/s, *p* = 0.01) compared with those without erectile dysfunction. There was also evidence of a marked small-fibre neuropathy with an impaired cold threshold (19.7 ± 1.4°C vs 27.3 ± 1.8°C, *p* = 0.003), warm threshold (42.9 ± 0.8°C vs 39.0 ± 0.9°C, *p* = 0.005) and heart rate variability (21.5 ± 3.1 vs 30.0 ± 3.7 beats/min, *p* = 0.001) and reduced intraepidermal nerve fibre density (2.8 ± 0.7 vs 5.9 ± 0.7/mm, *p* = 0.008), corneal nerve fibre density (12.6 ± 1.5 vs 23.9 ± 2.0/mm^2^, *p* < 0.001), corneal nerve branch density (12.7 ± 2.5 vs 31.6 ± 3.3/mm^2^, *p* < 0.001) and corneal nerve fibre length (8.3 ± 0.7 vs 14.5 ± 1.0 mm/mm^2^, *p* < 0.001) in participants with type 1 diabetes and erectile dysfunction. Erectile dysfunction correlated significantly with measures of both large- and small-fibre neuropathy.

**Conclusions/interpretation:**

Small-fibre neuropathy is prominent in patients with type 1 diabetes, and is associated with erectile dysfunction and can be objectively quantified using corneal confocal microscopy. This may allow the identification of patients who are less likely to respond to conventional therapies such as phosphodiesterase type 5 inhibitors.

## Introduction

Erectile dysfunction in patients with type 1 diabetes mellitus poses a major clinical problem and was associated with poorer diabetes-related quality of life in the DCCT/Epidemiology of Diabetes Interventions and Complications (EDIC) cohort, particularly in those with other complications including neuropathy [1]. It is principally mediated by impaired cavernosal vasodilatation due to a non-adrenergic, non-cholinergic nerve signalling defect, penile endothelial dysfunction and veno-occlusive disease; however, the relative contributions of each may differ between type 1 and type 2 diabetes [2].

Most earlier studies have primarily reported on erectile dysfunction in men with type 2 diabetes, and have demonstrated abnormalities in quantitative sensory testing (QST) results and sympathetic skin responses [3–7]. Recent studies in participants with type 1 diabetes from the DCCT and EDIC cohorts have also shown that cardiovascular autonomic neuropathy and peripheral neuropathy are major risk factors for erectile dysfunction [8, 9]. Furthermore, failure of erectile dysfunction therapy has been attributed to severe erectile dysfunction at presentation, worsening of endothelial dysfunction and the presence of a significant neuropathy [10, 11].

QST can identify small-fibre neuropathy; however, its subjective nature and high variability has limited its wider use. The Neuropathic Pain Specialist Interest Group consensus statement on QST cautions on the interpretation of results in relation to the clinical context [12]. More objective measures of small-fibre neuropathy include skin biopsy with assessment of intraepidermal nerve fibre density (IENFD), but this procedure is invasive, requires considerable laboratory expertise for analysis and has not been evaluated in patients with erectile dysfunction. Corneal confocal microscopy (CCM) is a rapid, non-invasive ophthalmic examination technique that objectively evaluates small-fibre neuropathy in patients with diabetes [13, 14] and is comparable with skin biopsy in the diagnosis of diabetic neuropathy [15, 16].

In this study, we undertook a comprehensive assessment of small- and large-fibre neuropathy to delineate the neuropathy status of an unselected cohort of men with type 1 diabetes in relation to erectile dysfunction.

## Methods

### Participant selection

We assessed 70 consecutive men with type 1 diabetes from the Central Manchester University Hospital Diabetes Centre and 34 age-matched healthy control participants. No formal power calculations could be undertaken as there are no previous studies evaluating small-fibre damage using IENFD or CCM in men with erectile dysfunction. The control group comprised healthy volunteers without diabetes mellitus who were taking no regular medication for any other comorbidity. These participants were relatives and friends of the study participants or University of Manchester staff, students and their relatives and friends. Men with type 1 diabetes were recruited and assessed from January 2009 to July 2014. All participants completed the study. Exclusion criteria were any history of neuropathy due to a non-diabetic cause, current or active diabetic foot ulceration, and any history of corneal trauma or surgery or a history of ocular disease or of a systemic disease that might affect the cornea. The Central Manchester Research and Ethics Committee approved this study, and written informed consent was obtained from all individuals prior to participation. This research adhered to the tenets of the declaration of Helsinki.

### Erectile dysfunction

Patients were assessed using the Neuropathy Symptom Profile (NSP), which specifically defines erectile dysfunction as the ‘inability to have sexual erection which is not due to medication or prostate surgery’ [17].

### Assessment of neuropathy

All study participants underwent assessment of BMI, BP, HbA_1c_, lipid profile (total cholesterol, LDL-cholesterol, HDL-cholesterol and triacylglycerol) and eGFR (calculated by the abbreviated Modification of Diet in Renal Disease [MDRD] equation: 186 × (creatinine / 88.4)^−1.154^ × (age)^−0.203^ × (0.742 if female) × (1.210 if black). The NSP was used to assess the symptoms of peripheral neuropathy. Neurological deficits were evaluated using the modified Neuropathy Disability Score, which is comprised of vibration perception, pinprick, temperature sensation and the presence or absence of ankle reflexes [18]. The vibration perception threshold (VPT) was tested using a Horwell Neurothesiometer (Scientific Laboratory Supplies, Wilfrod, Nottingham, UK). Cold (CT) and warm (WT) perception thresholds were established on the dorsolateral aspect of the left foot (S1) using the TSA-II NeuroSensory Analyser (Medoc, Ramat-Yishai, Israel). Electrodiagnostic studies were undertaken using a Dantec Keypoint system (Dantec Dynamics, Bristol, UK) equipped with a DISA temperature regulator to keep the limb temperature constantly at 32–35°C. Sural sensory nerve amplitude, sural sensory nerve conduction velocity, sural sensory nerve latency, peroneal motor nerve amplitude, peroneal motor nerve latency and peroneal motor nerve conduction velocity were assessed by a consultant neurophysiologist. The motor nerve study was performed using silver/silver chloride surface electrodes at standardised sites defined by anatomical landmarks, and recordings for the sural sensory nerve were taken using antidromic stimulation over a distance of 100 mm. Deep breathing heart rate variability (DB-HRV) was assessed using an ANX 3.0 autonomic nervous system monitoring device (ANSAR Medical Technologies, Philadelphia, PA, USA).

### Skin biopsy

A 3 mm punch skin biopsy was taken from the dorsum of the foot, approximately 2 cm above the second metatarsal head, under local anaesthesia (1% lidocaine). Sections (50 μm) were stained using anti-human PGP9.5 antibody (Abcam, Cambridge, UK) and nerve fibres were demonstrated using SG chromogen (Vector Laboratories, Peterborough, UK). IENFD was quantified in accordance with established criteria and expressed as number per millimetre [16].

### CCM

Patients underwent a CCM examination (Heidelberg Retinal Tomograph III Rostock Cornea Module; Heidelberg Engineering, Heidelberg, Germany) as per our previously established protocol [19]. Six non-overlapping images per participant (three per eye) from the centre of the cornea were selected and quantified in a masked fashion. Three corneal nerve variables were quantified: corneal nerve fibre density (CNFD; the total number of major nerves per square millimetre of corneal tissue), corneal nerve branch density (CNBD; the number of branches emanating from the major nerve trunks per square millimetre of corneal tissue) and corneal nerve fibre length (CNFL; the total length of all nerve fibres and branches [millimetre per square millimetre] within the area of corneal tissue). Analysis of corneal nerve morphology was performed using automated software, ACCMetrics (Manchester, UK) [20].

### Statistical analysis

Analysis was carried out using SPSS for Mac (Version 19.0; IBM Corporation, New York, NY, USA). All data are expressed as means ± SEM. Data were tested for normality using the Shapiro–Wilk normality test and by visualising the histogram and normal Q-Q plot. To assess within- and between-group differences, we used one-way ANOVA (non-parametric, Kruskal–Wallis test). ANCOVA was used to make age-adjusted comparisons between participants with type 1 diabetes with and without erectile dysfunction. A significant *p* value was considered to be <0.05 (post hoc; Tukey’s test).

## Results

All participants underwent all assessments except for skin biopsy, which was performed in 40 participants with type 1 diabetes and 21 control participants.

### Control participants vs men with type 1 diabetes

Participants in the control group were age matched to those with type 1 diabetes (45.4 ± 2.6 vs 46.2 ± 1.7 years, *p* = 0.77). The prevalence of erectile dysfunction was 58.6% in men with type 1 diabetes, compared with 5.9% in age-matched control participants. There were no differences in BMI, BP, smoking or alcohol consumption between the two groups. Participants with type 1 diabetes had a significantly higher HbA_1c_ level (7.7 ± 0.2% [58.9 ± 2.1 mmol/mol] vs 5.6 ± 0.1% [37.9 ± 0.7 mmol/mol], *p* < 0.0001) and lower total cholesterol (4.2 ± 0.1 vs 5.1 ± 0.1 mmol/l, *p* < 0.0001) and LDL-cholesterol (2.1 ± 0.1 vs 2.9 ± 0.1 mmol/l, *p* < 0.001) levels compared with control participants (Table 1). Patients with type 1 diabetes also had significantly higher NSP scores (3.9 ± 0.7 vs 0.2 ± 0.1, *p* < 0.001), Neuropathy Disability Scores (3.6 ± 0.4 vs 0.7 ± 0.2, *p* < 0.0001) and VPT (16.4 ± 1.6 vs 6.2 ± 0.9 V, *p* < 0.001) and lower sural nerve amplitude (7.5 ± 1.0 vs 17.9 ± 1.5 mV, *p* < 0.001), sural nerve conduction velocity (39.7 ± 1.1 vs 49.0 ± 0.6 m/s, *p* < 0.001), peroneal nerve amplitude (3.1 ± 0.4 vs 6.2 ± 0.3 mV, *p* < 0.001) and peroneal nerve conduction velocity (37.5 ± 1.2 vs 48.8 ± 0.7 m/s, *p* < 0.001) compared with control participants (Table 2). Furthermore, individuals with type 1 diabetes had a significantly higher WT (41.4 ± 0.6°C vs 37.6 ± 0.7°C, *p <* 0.001) and a significantly lower CT (22.7 ± 1.0°C vs 28.2 ± 0.4°C, *p* < 0.001), DB-HRV (25.1 ± 2.4 vs 31.0 ± 2.2 beats/min, *p* < 0.001), IENFD (4.3 ± 0.5 vs 10.5 ± 0.7/mm, *p* < 0.001), CNFD (16.9 ± 1.2 vs 30.1 ± 1.2/mm^2^, *p* < 0.001), CNBD (19.8 ± 2.0 vs 37.1 ± 2.7/mm^2^, *p* < 0.001) and CNFL (10.7 ± 0.6 vs 17.1 ± 0.6 mm/mm^2^, *p* < 0.001), compared with control participants (Table 2).

**Table 1:** Background demographic factors and clinical variables for control participants vs participants with type 1 diabetes and no erectile dysfunction vs participants with type 1 diabetes and erectile dysfunction

Characteristic	Control participants (*n* = 34)	Type 1 diabetes, no ED (*n* = 29)	Type 1 diabetes, ED (*n* = 41)	*p* value
Age (years)	45.4 ± 2.6	41.8 ± 2.3	57.1 ± 1.9	
BP, systolic/diastolic (mmHg)	136.9 ± 3.0/75.2 ± 1.8	133 ± 3.1/70.5 ± 1.9	139 ± 3.9/73.2 ± 1.5	0.8/0.7
HbA_1c_ (mmol/mol)	37.9 ± 0.7***	62.3 ± 2.8	58.8 ± 3.0	0.7
HbA_1c_ (%)	5.6 ± 0.1***	7.9 ± 0.3	7.6 ± 0.3	
Duration of diabetes (years)^a^	–	28.8 ± 2.3	28.1 ± 1.8	0.8
BMI (kg/m^2^)	26.4 ± 0.6	26.8 ± 0.9	26.5 ± 0.7	0.7
Albumin/creatinine ratio (mg/mmol)	0.6 ± 0.3*	0.8 ± 0.2	6.4 ± 3.3	<0.001
eGFR (ml min^−1^ [1.73 m]^−2^)	85.2 ± 1.2	87.4 ± 1.4	66.6 ± 3.7	<0.001
Smoking (cigarettes/day)	0.3 ± 0.3	0.9 ± 0.6	1.2 ± 0.7	0.4
Alcohol (units/week)	6.9 ± 1.9	3.8 ± 1.4	7.2 ± 1.9	0.4
Total cholesterol (mmol/l)	5.1 ± 0.1***	4.2 ± 0.2	4.1 ± 0.2	0.8
HDL-cholesterol (mmol/l)	1.4 ± 0.1	1.5 ± 0.1	1.5 ± 0.1	0.6
Triacylglycerol (mmol/l)	1.5 ± 0.1*	1.3 ± 0.2	1.2 ± 0.1	0.9
LDL-cholesterol (mmol/l)	2.9 ± 0.1***	2.1 ± 0.2	2.1 ± 0.1	0.9
ED, yes (%)	5.9	58.6 (all participants with diabetes)		

Data are means ± SEM unless otherwise stated

^a^Adjusted for age using ANCOVA

*p < 0.05, ***p < 0.001 control participants vs men with type 1 diabetes mellitus

*p* value is for comparison between participants with and without erectile dysfunction

ED, erectile dysfunction

**Table 2:** Neuropathy assessments for control participants vs participants with type 1 diabetes mellitus and no erectile dysfunction vs type 1 diabetes and erectile dysfunction

Variable	Control participants (*n* = 34)	Type 1 diabetes, no ED (*n* = 29)	Type 1 diabetes, ED (*n* = 41)	*p* value
NSP (/38)^a^	0.2 ± 0.1***	1.8 ± 1.2	5.3 ± 0.9	0.03
Neuropathy Disability Score (/10)^a^	0.7 ± 0.2***	2.8 ± 0.7	4.1 ± 0.6	0.1
VPT (V)^a^	6.2 ± 0.9***	10.7 ± 2.4	18.3 ± 1.9	0.02
Sural nerve amplitude (μV)^a^	17.9 ± 1.5***	11.7 ± 1.5	5.0 ± 1.1	0.002
Sural nerve conduction velocity (m/s)^a^	49.0 ± 0.6***	42.6 ± 1.9	37.9 ± 1.4	0.07
Peroneal nerve amplitude (mV)^a^	6.2 ± 0.3***	4.7 ± 0.5	2.1 ± 0.4	<0.001
Peroneal nerve conduction velocity (m/s)^a^	48.8 ± 0.7***	41.9 ± 2.0	34.8 ± 1.5	0.01
CT (°C)^a^	28.2 ± 0.4***	27.3 ± 1.8	19.7 ± 1.4	0.003
WT (°C)^a^	37.6 ± 0.7***	39.0 ± 0.9	42.9 ± 0.8	0.005
IENFD (*n*/mm)^a^	10.5 ± 0.7***	5.9 ± 0.7	2.8 ± 0.7	0.008
Automated CNFD (*n*/mm^2^)^a^	30.1 ± 1.2***	23.9 ± 2.0	12.6 ± 1.5	<0.001
Automated CNBD (*n*/mm^2^)^a^	37.1 ± 2.7***	31.6 ± 3.3	12.7 ± 2.5	<0.001
Automated CNFL (mm/mm^2^)^a^	17.1 ± 0.6***	14.5 ± 1.0	8.3 ± 0.7	<0.001
DB-HRV (beats/min)^a^	31.0 ± 2.2***	30.0 ± 3.7	21.5 ± 3.1	0.001

Data are means ± SEM

^a^Adjusted for age using ANCOVA

***p < 0.001, control participants vs men with type 1 diabetes mellitus

*p* value is for comparison between participants with and without erectile dysfunction

ED, erectile dysfunction

### Type 1 diabetes participants with and without erectile dysfunction

Type 1 diabetes participants without erectile dysfunction were younger than those with erectile dysfunction (41.8 ± 2.3 vs 57.1 ± 1.85 years) (Table 1). There were no differences in BP, BMI, HbA_1c_ and lipid profile between the two groups, but eGFR was significantly lower and the albumin/creatinine ratio significantly higher (both *p* < 0.001) in participants with erectile dysfunction. After adjusting for age, both groups had a comparable HbA_1c_ level. Patients with type 1 diabetes and erectile dysfunction had a higher NSP score (5.3 ± 0.9 vs 1.8 ± 1.2, *p* = 0.03) and VPT (18.3 ± 1.9 vs 10.7 ± 2.4 V, *p* = 0.02), and a lower sural nerve amplitude (5.0 ± 1.1 vs 11.7 ± 1.5 mV, *p* = 0.002), peroneal nerve amplitude (2.1 ± 0.4 vs 4.7 ± 0.5 mV, *p* < 0.001) and peroneal nerve conduction velocity (34.8 ± 1.5 vs 41.9 ± 2.0 m/s, *p* = 0.01) compared with participants without erectile dysfunction (Table 2).

**Fig. 1:**
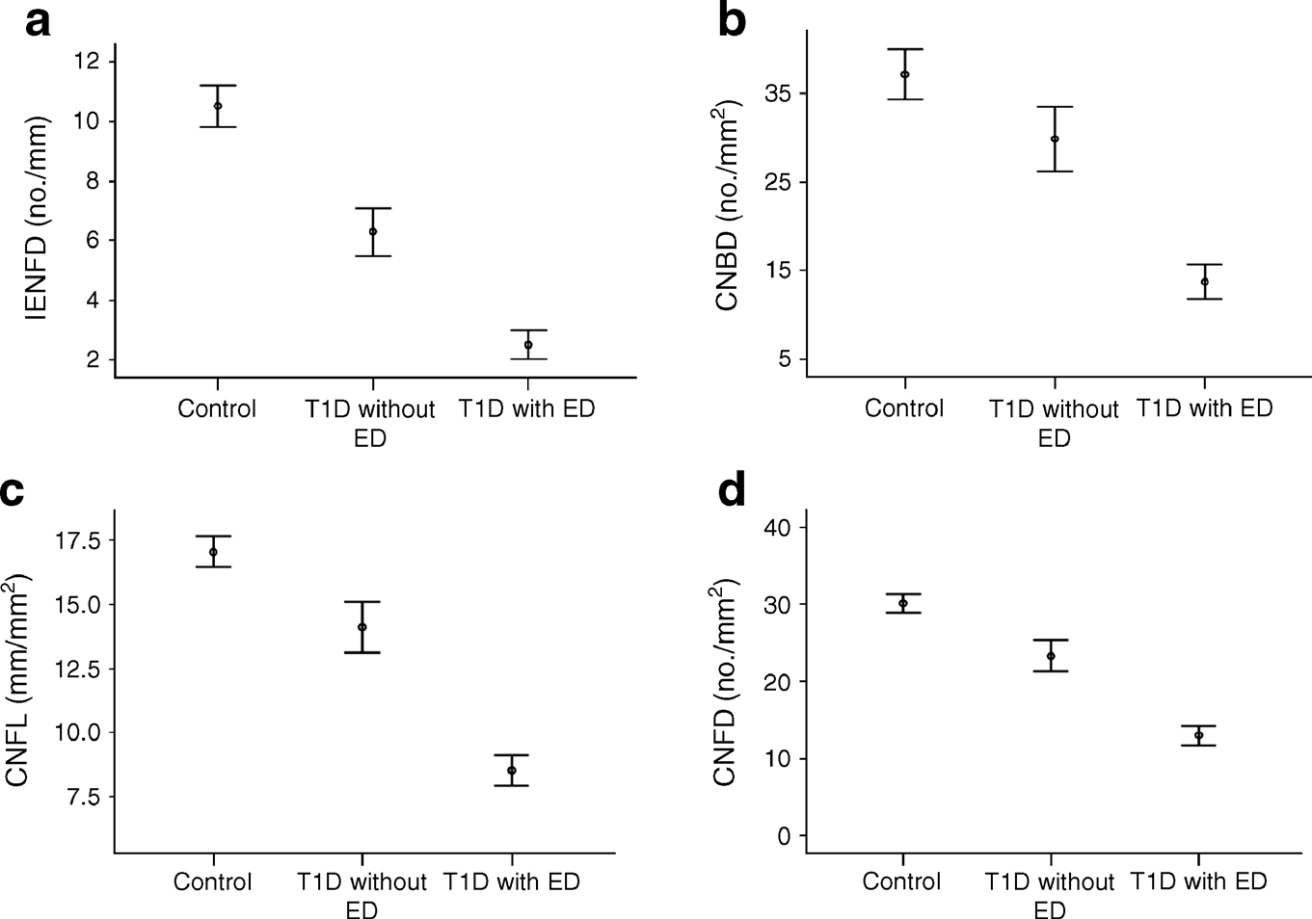
IENFD (**a**) and CCM (**b**–**d**) data from control participants, men with type 1 diabetes mellitus with normal erectile function and men with type 1 diabetes mellitus with erectile dysfunction. Data are means ± SEM. ED, erectile dysfunction; T1D, type 1 diabetes

**Fig. 2:**
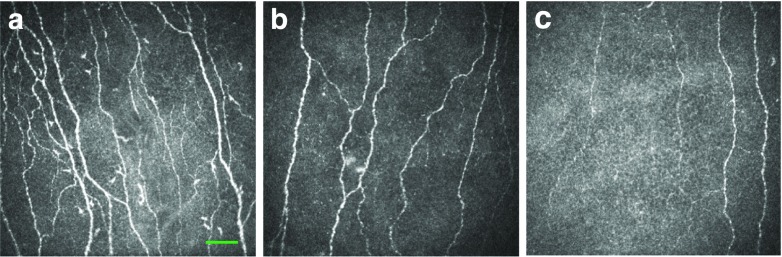
CCM images of the corneal sub-basal nerves of: (**a**) a control participant; (**b**) a participant with type 1 diabetes mellitus and no erectile dysfunction; and (**c**) a participant with type 1 diabetes mellitus and erectile dysfunction. Scale bar, 50 μm

WT (42.9 ± 0.8°C vs 39.0 ± 0.9°C, *p* = 0.005) was significantly higher in participants with erectile dysfunction compared with those without, while CT (19.7 ± 1.4°C vs 27.3 ± 1.8°C, *p* = 0.003), DB-HRV (21.5 ± 3.1 vs 30.0 ± 3.7 beats/min, *p* = 0.001), IENFD (2.8 ± 0.7 vs 5.9 ± 0.7/mm, *p* = 0.008), CNFD (12.6 ± 1.5 vs 23.9 ± 2.01/mm^2^, *p* < 0.001), CNBD (12.7 ± 2.5 vs 31.6 ± 3.3/mm^2^, *p* < 0.001) and CNFL (8.3 ± 0.7 vs 14.5 ± 1.0 mm/mm^2^, *p* < 0.001) were all significantly lower (Figs 1, 2).

Erectile dysfunction correlated significantly with NSP (*r* = 0.561, *p* < 0.001), Neuropathy Disability Score (*r* = 0.452, *p* < 0.001), VPT (*r* = 0.619, *p* < 0.001), CT (*r* = −0.488, *p* < 0.001), WT (*r* = 0.496, *p* < 0.001), sural nerve amplitude (*r* = −0.655, *p* < 0.001), sural nerve conduction velocity (*r* = −0.548, *p* < 0.001), peroneal nerve amplitude (*r* = −0.685, *p* < 0.001), peroneal nerve conduction velocity (*r* = −0.635, *p* < 0.001), IENFD (*r* = −0.603, *p* < 0.001), CNFD (*r* = −0.641, *p* < 0.001), CNBD (*r* = −0.552, *p* < 0.001) and CNFL (*r* = −0.657, *p* < 0.001).

There were no correlations between erectile dysfunction and duration of diabetes (*r =* 0.012, *p =* 0.354)*, BMI* (*r* = −0.011, *p* = 0.926), BP (systolic, *r* = 0.025, *p* = 0.828; diastolic, *r* = −0.004, *p* = 0.975), HbA_1c_ (*r* = −0.174, *p* = 0.169), total cholesterol (*r* = 0.020, *p* = 0.874), HDL-cholesterol (*r* = −0.051, *p* = 0.689), LDL-cholesterol (*r* = 0.001, *p* = 0.994) or triacylglycerol (*r* = −0.004, *p* = 0.978).

## Discussion

In this study, we have shown a high prevalence of erectile dysfunction in men with type 1 diabetes mellitus, and demonstrated large- and particularly small-fibre and autonomic neuropathy in men with erectile dysfunction. The majority of previous prevalence studies of erectile dysfunction have not distinguished between type 1 and type 2 diabetes, and have in fact focused primarily on individuals with type 2 diabetes [21]. However, data from the UroEDIC study showed that 55% of men with type 1 diabetes had decreased libido and 34% suffered from erectile dysfunction [22]. In another study of men with type 1 diabetes mellitus, the self-reported erectile dysfunction prevalence was 47.1% among those aged 43 years or older [23]. Age and the duration of diabetes may affect the prevalence of erectile dysfunction and, of course, differences in diagnosing erectile dysfunction and in population characteristics may also be partly responsible for the variability in reported prevalence rates, which range from 35% to 75% [21, 24]. While the duration of diabetes, poor glycaemic control, hypertension, hyperlipidaemia and obesity have previously been associated with erectile dysfunction in men with type 2 diabetes [25], our study in type 1 diabetes did not find a correlation between erectile dysfunction and HbA_1c_, BMI, hypertension or duration of diabetes. The long duration of diabetes in our study population and the use of a single HbA_1c_ measurement, as opposed to an average life-time value, limit the relevance of this study to a wider population of men with type 1 diabetes. Nonetheless, the long duration of diabetes and the age of the men in this study are typical of those at greatest risk of erectile dysfunction.

Although erectile dysfunction has previously been shown to correlate with age and the presence of symptomatic peripheral and autonomic neuropathy [23, 24], vascular function has been investigated more often than neuropathy as a means of identifying patients who may be more or less responsive to treatment. In men with peripheral neuropathy, sensory impulses from the shaft and glans of the penis to the reflexogenic erectile centre and pudendal nerve innervation of the pelvic floor muscles are impaired. This limits contraction of the bulbocavernous and ischiocavernosus muscles, which normally contribute to reduced venous outflow from the cavernous bodies and maintenance of an erection [21]. As parasympathetic activity is involved in achieving an erection, autonomic neuropathy is strongly associated with erectile dysfunction [21]. Furthermore, nitric oxide plays a key role in maintaining penile erection, and is synthesised and released via both the endothelium and autonomic nerves of the penile arteries and corpus cavernosum [26].

Certain populations are less responsive to phosphodiesterase type 5 (PDE5) inhibitor therapy, which is the first-line treatment in the management of erectile dysfunction [27]. These include patients with severe diabetic neuropathy, and those with neurological damage from procedures such as radical prostatectomy and severe vascular disease [11, 27]. PDE5 inhibitors require a minimum amount of nitric oxide production, which is not possible with severely damaged nerves. It has been suggested that therapeutic strategies to promote nitric oxide synthesis and availability may improve erectile function and increase the effectiveness of PDE5 inhibitors in patients who are currently less responsive to such therapies [27].

In a large study of 341 participants with erectile dysfunction, peripheral neuropathy was identified in 38% of individuals with diabetes and 10% of non-diabetic participants using nerve conduction studies and QST [4]. Among those individuals who had a vasculogenic basis for their erectile dysfunction, the nocturnal tumescence test indicated that the majority also had neuropathy [4]. Others researchers have found impaired thermal thresholds, capsaicin-induced sensory axon-reflex vasodilatation and sural nerve amplitude in men with erectile dysfunction [5, 6]. Bleustein et al. undertook QST of the penis and showed that non-diabetic participants with erectile dysfunction had impaired thermal thresholds and VPTs, and that participants with type 1 diabetes and erectile dysfunction had a large- and small-fibre neuropathy [7]. This is consistent with our finding of significant large- and small-fibre neuropathy in participants with type 1 diabetes with erectile dysfunction compared with those without erectile dysfunction and control participants. More specific neurological evaluations for erectile dysfunction can include measuring the bulbocavernosus reflex, penile thermal sensory thresholds and somatosensory evoked potentials, and conducting corpus cavernosum electromyography. However, these are highly specialised tests that lack reproducibility, with no age-adjusted normal values to aid in diagnosis. Nonetheless, the central role of small-fibre dysfunction is evidenced by a previously reported strong correlation between penile thermal sensory testing and the clinical evaluation of erectile dysfunction [28], and by a lack of correlation between neurophysiology and erectile dysfunction severity, as determined using the International Index of Erectile Function [8].

In the current study, we have demonstrated widespread small-fibre damage, as evidenced by a reduction in IENFD in foot skin biopsies and the observation of corneal nerve fibre abnormalities using CCM in participants with type 1 diabetes and erectile dysfunction. Indeed, we have previously demonstrated the very high sensitivity and specificity of CCM in identifying diabetic autonomic neuropathy [29]. Furthermore, IENFD and CCM abnormalities correlated with erectile dysfunction. However, IENFD is invasive and cannot be routinely deployed in the diagnostic work-up of patients with erectile dysfunction. CCM, on the other hand, is a non-invasive objective method with which to quantify small-fibre damage, using an unbiased automated image analysis technique [30, 31] that has previously been reported to correlate with IENFD [15] and, in the present study, correlated significantly with erectile dysfunction. However, a range of small-fibre abnormalities are seen in patients with erectile dysfunction. Therefore, the next step would be to assess whether patients with more severe small-fibre damage are less likely to respond to therapy for erectile dysfunction.

The diagnosis and management of erectile dysfunction in men with diabetes is challenging, with a greater failure rate of erectile dysfunction therapies than in the non-diabetic population [32]. The identification of more extensive small-fibre damage using CCM may allow us to identify those patients with erectile dysfunction who are less likely to respond to conventional therapies such as PDE5 inhibitors, and who should therefore be considered for daily or higher doses of PDE5 inhibitors, combination therapies or, indeed, alternative therapies such as intraurethral alprostadil or a penile prosthesis [33, 34].

## Abbreviations

CCM: Corneal confocal microscopy
CNBD: Corneal nerve branch density
CNFD: Corneal nerve fibre density
CNFL: Corneal nerve fibre length
CT: Cold perception threshold
DB-HRV: Deep breathing heart rate variability
EDIC: Epidemiology of Diabetes Interventions and Complications
IENFD: Intraepidermal nerve fibre density
NSP: Neuropathy symptom profile
PDE5: Phosphodiesterase type 5
QST: Quantitative sensory testing
VPT: Vibration perception threshold
WT: Warm perception threshold
